# Telomere Length, HLA, and Longevity—Results from a Multicenter Study

**DOI:** 10.3390/ijms25179457

**Published:** 2024-08-30

**Authors:** Marta Dratwa-Kuzmin, Bushra Al Hadra, Fatma Oguz, Yeliz Ogret, Ileana Constantinescu, Dimitri Apostol, Adriana Talangescu, Alexandra-Elena Constantinescu, Ion Maruntelu, Katarzyna Kościńska, Tsvetelin Lukanov, Elissaveta Naumova, Katarzyna Bogunia-Kubik

**Affiliations:** 1 Laboratory of Clinical Immunogenetics and Pharmacogenetics, Hirszfled Institute of Immunology and Experimental Therapy, Polish Academy of Sciences, 53-114 Wroclaw, Poland; marta.dratwa-kuzmin@hirszfeld.pl; 2 Clinic of Clinical Immunology and Stem Cell Bank, University Hospital Alexandrovska, 1431 Sofia, Bulgaria; bushraalkhadra@gmail.com (B.A.H.); ts_lukanov@yahoo.com (T.L.); naumovaej@gmail.com (E.N.); 3 Department of Clinical Immunology, Medical University, 1431 Sofia, Bulgaria; 4 Department of Medical Biology, Istanbul Medical Faculty, Istanbul University, 34098 Istanbul, Turkey; oguzsf@gmail.com (F.O.); yelizdogret@gmail.com (Y.O.); 5 Centre for Immunogenetics and Virology, Fundeni Clinical Institute, Carol Davila University of Medicine and Pharmacy, 022328 Bucharest, Romania; ileana.constantinescu@imunogenetica.ro (I.C.); apostol_dimitri@yahoo.com (D.A.); drianaoprea1989@yahoo.com (A.T.); alexandra-elena.constantinescu0720@stud.umfcd.ro (A.-E.C.); ion.maruntelu@drd.umfcd.ro (I.M.); 6 HLA Laboratory, Lower Silesian Oncology, Pulmonology and Hematology Center, 54-049 Wroclaw, Poland; katarzyna.koscinska@dcopih.pl

**Keywords:** telomere length, HLA, longevity, aging

## Abstract

Aging is an exceptionally complex process that depends on genetic, environmental, and lifestyle factors. Previous studies within the International HLA and Immunogenetics Workshop (IHIWS) component “Immunogenetics of Ageing” showed that longevity is associated with positive selection of HLA-DRB1*11- and DRB1*16-associated haplotypes, shown to be protective against diseases. Within the 18th IHIWS, we aimed to investigate the relevance of telomere length for successful aging and its association with classical HLAs. In total 957 individuals from Bulgaria, Turkey, Romania, and Poland in two age groups, elderly individuals (age 65–99 years) and ethnically matched young group (age 18–64 years), were investigated. The obtained results confirmed interpopulation differences in the distribution of HLA alleles, documented the lengths of telomeres in analyzed populations, and demonstrated significant associations of telomere length with aging as well as with the presence of some HLA class I or class II alleles. They suggest that telomere length assessment combined with HLA genotyping may help identify immunogenetic profiles associated with longevity. The associations between HLA and telomeres support the theory that HLA genes influence the aging process. However, further research is needed to clarify the biological basis of the observed relationships.

## 1. Introduction

Aging is a complex biological process that involves the accumulation of abnormalities in cellular processes, signaling, and regulatory mechanisms, ultimately leading to the disruption of tissue homeostasis [1]. While its biological causes remain largely unknown, recent studies have identified several hallmarks of aging. Hayflick and Moorhead first described cellular senescence as a progressive and irreversible loss of replication capacity of human somatic cells, accompanied by a series of changes in cell morphology, gene expression, metabolism, and epigenetics [2].

In 1986, Cooke and Smith published the first article regarding the relationship between telomere length and cell aging [3]. Lately, it was discovered that the replication capacity of human cells depends on the action of the telomerase enzyme, which has a crucial role in maintaining the length of telomeres [4,5].

In recent years, it has been suggested that telomere length may be a potential cellular marker of biological aging, due to the inverse relationship between chronological age and telomere length. Mean telomere length shows considerable interindividual variability and has a high heritability, with an estimated value of 44–80% [6,7]. The length of telomere decreases with age by an average of 20–50 nucleotides per year [8]. However, telomere shortening is not a linear process where a constant number of base pairs is lost with every cell division [9]. The value of telomere length at any one time depends on genetic characteristics and the balance between “shortening” and “elongation” signals [10]. Interestingly, short telomeres have been identified as an independent risk factor for worsening of function in older European populations [11]. This situation may be related to altered fatty acid metabolism and increased oxidative stress in the process of human aging [12]. Moreover, gender and ethnicity were found to be significantly associated with telomere length. In general, older adults have shorter telomeres than younger adults, and women who live longer than men have longer telomeres on average [13]. Additionally, African or Hispanic ancestry has been linked to a predisposition to having longer telomeres compared to being male and European origin [14,15]. A study monitoring telomere length from birth to adulthood found a correlation between the effects of prenatal exposures and maternal conditions, such as obstetric complications, body mass index (BMI), nutritional status, stress, and sociological status during pregnancy [16].

The major histocompatibility complex (MHC) is traditionally divided into the class I (HLA-A, -B, and -C), class II (HLA-DR, -DQ, and -DP), and class III regions. The immune response depends on various factors, the most important of which is the ability of human leukocyte antigens (HLAs) to bind some peptides and not others [17]. Due to the important role of HLA in the development of human protective immunity, many studies have focused on the possible influence of these genes on human longevity.

The immunogenetics of aging and longevity is a complex concept. To date, only few studies have shown an imbalance in the distribution of HLA in the elderly compared to the young, and to the best of our knowledge, there are no publications on the potential associations between telomere length, HLA, and aging. Thus, the aim of this study was to assess and compare the telomere length between unrelated young and elderly representatives of Bulgaria, Turkey, Romania, and Poland and to explore the relationships between HLA class I (HLA-A, -B, and -C) and class II (HLA-DR, -DQ, and -DP) alleles/haplotypes and telomere length in unrelated elderly and young people from these four populations. Data for the analyses were collected by the Immunogenetics of Aging working group.

## 2. Results

### 2.1. Distribution of HLA Class I and II Alleles in the Studied Populations

The distribution of HLA class I and class II two-loci haplotypes were analyzed in Bulgarians, Romanians, Turks, and Poles and compared between elderly and young representatives of all four studied populations. Classic HLA haplotypes observed at statistically significant increased frequency in older adults from four populations compared to ethnically matched young groups are shown in Table 1, Table 2 and Table 3, respectively. As expected, some differences were noted among and between representatives of all studied groups. For example, HLA-A*02 was one of the most common alleles detected. However, its frequency varied between elderly and young individuals. It appeared that increased frequencies of A*02-associated haplotypes were observed among elderly Bulgarians and Turks, but lower frequencies in Romanians and Poles as compared to the respective young group.

### 2.2. Comparison of Telomere Length among Elderly and Young Representatives in the Studied Populations

**Figure 1:**
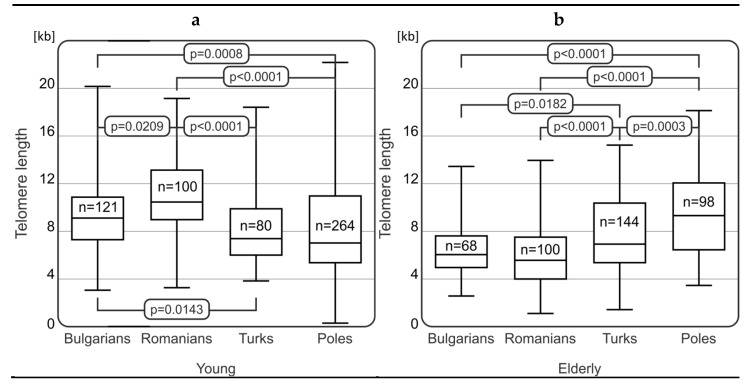
Differences in telomere length within young (**a**) and elderly (**b**) populations from Bulgaria, Romania, Turkey, and Poland.

The comparison of telomere length in the analyzed groups showed significant differences between the populations from Bulgaria, Romania, Turkey, and Poland (Figure 1). Among the elderly individuals, Poles (*n* = 98) had the longest telomeres (mean telomere length was 10.29 ± 3.94 kb) as compared to elderly group from Bulgaria (*n* = 68; mean telomere length was 6.51 ± 2.23 kb; *p* < 0.0001), Romania (*n* = 100; mean telomere length was 6.01 ± 2.57 kb; *p* < 0.0001), and Turkey (mean telomere length was 7.83 ± 3.47 kb; *p* < 0.0001) (Figure 1b). Additionally, significant differences in telomere length were observed between healthy-aged Turks and Bulgarians (*p* = 0.0182) as well as between Turks and Romanians (*p* < 0.0001) (Figure 1b).

While in young study groups (Figure 1a), Romanians (*n* = 100) had the longest telomere length (mean telomere length was 9.05 ± 4.35 kb) as compared to young individuals from Bulgaria (*n* = 121; mean telomere length was 7.89 ± 2.68 kb; *p* = 0.0209), Turkey (*n* = 80; mean telomere length was 6.93 ± 2.68 kb; *p* < 0.0001), and Poland (*n* = 264; mean telomere length was 7.41 ± 4.35; *p* < 0.0001). Moreover, some significant relationships were observed between young Bulgarians compared to young Turks (*p* = 0.0143) and young Poles (*p* = 0.0008).

No associations were found between telomere length and gender.

In the context of differences between young and elderly individuals within particular populations, it was generally expected that longer telomeres would predominate in younger people. However, we did not observe any significant differences in telomere length within the Turkish group (mean telomere length of 7.83 ± 3.47 vs. 6.93 ± 2.68 kb, for elderly vs. young Turks). As expected, significant differences in telomere length were observed between elderly and young groups in the two analyzed populations. Elderly Bulgarians and Romanians had shorter telomeres than those belonging to the younger groups (*p* < 0.0001, in all cases; Figure 1b). The differences in the means of telomere lengths were as follows for the elderly versus young groups: Bulgaria (6.51 ± 2.23 vs. 7.89 ± 2.68 kb) and Romania (6.01 ± 2.57 vs. 9.05 ± 4.35 kb; *p* < 0.0001). However, quite surprisingly, they were opposite for elderly versus young Poles (7.42 ± 4.35 vs. 10.29 ± 3.94, *p* < 0.0001).

### 2.3. Relationships between Telomere Length and HLA Genotype

**Figure 2:**
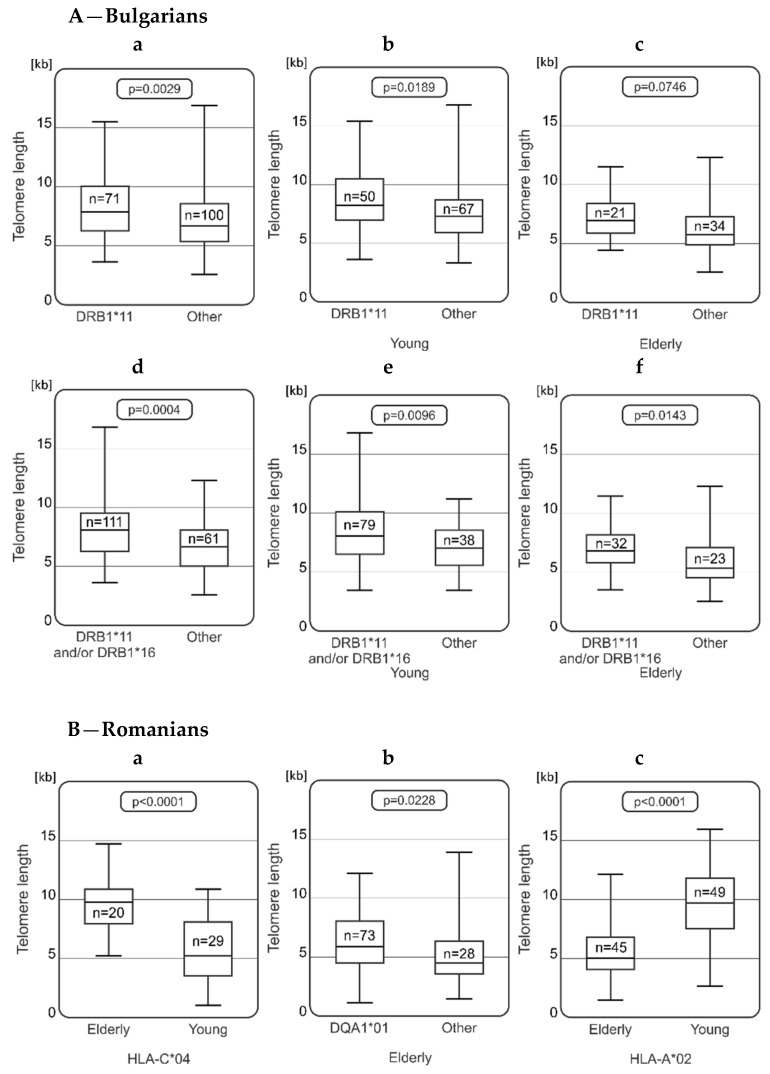

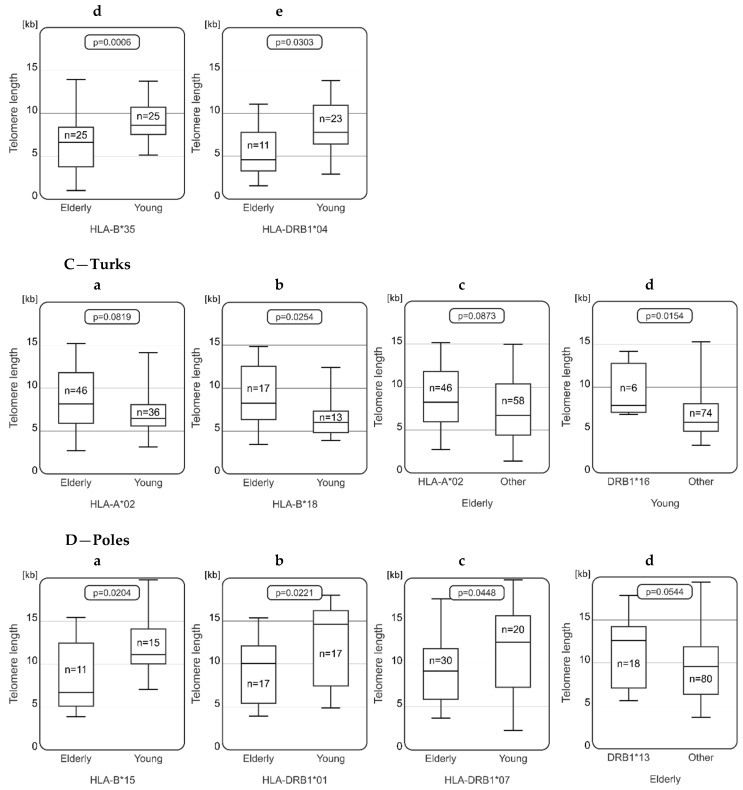
Significant associations between telomere length and HLA alleles, detected in elderly and young representatives of the studied populations. **A**—Bulgarians carrying HLA-DRB1*11 and/or DRB1*16 have significantly longer telomeres than individuals with other HLA-DRB1 alleles (**a**,**d**) and this relationship is observed in both young (**b**,**e**) and elderly Bulgarians (**c**,**f**). **B**—Among Romanians, longer telomeres were detected in the HLA-C*04-positive elderly group as compared to young group carrying HLA-C*04 (**a**), and in elderly Romanians with DQA1*01 as compared to elderly young Romanians lacking these DQA1 alleles (**b**); young Romanians carrying the HLA-A*02 (**c**), HLA-B*35 (**d**), and HLA-DRB1*04 (**e**) alleles, characterized by longer telomeres than elderly Romanians carrying these HLA alleles. **C**—Healthy elderly Turks carrying HLA-A*02 (**a**) and B*18 (**b**) had longer telomeres than the young group; longer telomers were also detected in A*02-positive than in A*02-negative elderly Turks (**c**) and in DRB1*16-positive than in DRB1*16-negative young representatives of the Turkish population (**d**). **D**—young Poles carrying HLA-B*15 (**a**), DRB1*01 (**b**), and DRB1*07 (**c**) have longer telomeres than healthy elderly Poles carrying these alleles; moreover, a tendency was observed for differences in telomere length among healthy elderly Poles carrying or lacking DRB1*13 (**d**). n—indicates the number of healthy elderly or young individuals carrying a given HLA allele/genotype.

In the Bulgarian population, an association between telomere length and the presence of HLA-DRB1 alleles was observed (Figure 2Aa). Longer telomeres were detected in Bulgarians carrying HLA-DRB1*11 and/or DRB1*16 alleles (Figure 2Ad). DRB1*11 was associated with longer telomere length in all studied Bulgarians (*n* = 71; mean telomere length was 8.25 ± 2.60 kb vs. 7.05 ± 2.49 kb; *p* = 0.0029). This relationship was observed in young Bulgarians (*n* = 50; 8.65 ± 2.73 kb vs. 7.47 ± 2.52 kb; *p* = 0.0189; (Figure 2Ab)), and the trend was noticed in elderly Bulgarians (*n* = 21; 7.29 ± 2.01 vs. 6.21 ± 2.21 kb; *p* = 0.0746; (Figure 2Ac)). Furthermore, Bulgarians carrying HLA-DRB1*11 and/or DRB1*16 were found with significantly longer telomeres than individuals with other HLA-DRB1 alleles (for all analyzed Bulgarians (Figure 2Ad), mean telomere length was 8.07 ± 2.68 kb, *p* = 0.0004; for the young group, 8.45 ± 2.83 kb vs. 6.97± 1.97 kb, *p* = 0.0096 (Figure 2Ae); and for the elderly, 7.13 ± 2.02 kb vs. 5.92 ± 2.24 kb, *p* = 0.0143 (Figure 2Af)).

The differences in the frequencies of HLA-DRB1-associated haplotypes in elderly and young Bulgarians are presented on Table 1, where DRB1*11- (A*01~DRB1*11, HF young: 0.0451, HF elderly: 0.1048, OR = 2.48, *p* = 0.030) and DRB1*16- (A*02~B*35~DRB1*16 HF young: 0, HF elderly: 0.0323, OR = 18.26, *p* = 0.005) associated haplotypes were found to prevail significantly among elderly Bulgarians (Table 1).

**Table 1:** Statistically significant (*p* < 0.05) frequency of HLA-DRB1-associated haplotypes in elderly and young Bulgarians.

Haplotype	HF Young (*n* = 122)	HF Elderly (*n* = 62)	OR	CI.L	CI.U	*p*-Value
A*02~B*35~DRB1*16	0	0.0323	18.26	0.98	341.97	0.005
A*02~B*49~DRB1*14	0	0.0242	14.09	0.72	274.91	0.015
A*02~B*51~DRB1*10	0	0.0161	9.98	0.48	209.50	0.047
A*02~B*57~DRB1*07	0	0.0161	9.98	0.48	209.50	0.047
A*24~B*40~DRB1*03	0	0.0161	9.98	0.48	209.50	0.047
A*01~DRB1*11	0.0451	0.1048	2.48	0.99	6.31	0.030
A*02~DRB1*10	0.0041	0.0323	8.10	0.90	73.27	0.030
B*35~DRB1*07	0.0041	0.0323	8.10	0.90	73.27	0.030
C*08~DRB1*01	0.0410	0	0.09	0.01	1.54	0.020
DRB1*04~DQB1*04	0	0.0242	14.09	0.72	274.91	0.015
DRB1*07~DQB1*03	0.0041	0.0323	8.10	0.90	73.27	0.030

HF: haplotype frequency; n: the number of individuals included in this study; OR: odds ratio; CI: confidence interval (95%). *p*-values ≤ 0.05 were considered statistically significant.

In Romanians, associations between HLA type and telomere length were also observed (Figure 2B). Among HLA-C*04-positive Romanians, longer telomeres were detected in the elderly group (*n* = 20) than in the young group (*n* = 29) (mean telomere length was 9.60 ± 2.26 vs. 5.88 ± 2.57 kb, *p* < 0.0001; Figure 2Ba). Additionally, analysis showed that younger people carrying HLA-A*02, B*35, and DRB1*04 have longer telomere lengths than their older counterparts (mean telomere length was 9.46 ± 3.16 vs. 5.69 ± 2.71 kb, *p* < 0.0001; 9.10 ± 2.36 vs. 6.26 ± 3.07 kb, *p* = 0.0006; 8.36 ± 3.28 vs. 5.62 3.32 kb, *p* = 0.0303; Figure 2Bc, Figure 2Bd, and Figure 2Be, respectively). Higher frequencies of HLA-A*02, B*35, and DRB1*04 associated haplotypes in younger Romanians were observed as well (Table 2). Furthermore, a relationship was observed between HLA class II DQA1 variants and length of telomeres. Among older Romanians, individuals carrying HLA-DQA1*01 (*n* = 73) had longer telomeres than Romanians with other HLA-DQA1 alleles (*n* = 28) (mean telomere length 6.28 ± 2.53 kb vs. 5.18 ± 2.58 kb, *p* = 0.0028; (Figure 2Bb). Of note, HLA-DQA1*01-associated haplotype was observed with an increased frequency in elderly Romanians (HF young: 0.025, HF elderly: 0.065, OR = 2.71, *p* = 0.054; Table 2).

**Table 2:** Classical HLA haplotypes observed with significantly increased frequency (*p* < 0.05) in elderly and young individuals from the Romanian population.

Haplotype	HF Young *(n* = 100)	HF Elderly (*n* = 100)	OR	CI.L	CI.U	*p*-Value
A*02~DRB1*12	0.03	0	0.07	0.00	1.33	0.030
A*03~DRB1*01	0.005	0.04	8.29	1.03	66.93	0.040
A*01~B*08	0.02	0.07	3.69	1.13	15.62	0.016
A*02~B*35	0.075	0.03	0.38	0.12	1.07	0.040
A*02~C*07	0.14	0.07	0.46	0.22	0.95	0.020
A*68~C*07	0	0.04	17.71	1.01	308.89	0.007
DRB1*04~DQB1*03	0.125	0.065	0.49	0.22	1.03	0.040
DRB1*04~DQA1*03~DQB1*03	0.125	0.055	0.41	0.18	0.89	0.010
DRB1*14~DQA1*01~DQB1*05	0.025	0.065	2.71	0.88	9.88	0.054
A*01~B*08~DRB1*03	0.015	0.055	3.82	0.99	21.6	0.030

HF: haplotype frequency; n: the number of individuals included in this study; OR: odds ratio; CI: confidence interval (95%). *p*-values ≤ 0.05 were considered statistically significant.

Some relationships between telomere length and HLA type were also observed in Turks (Figure 2C). As within the whole Turkish population, also among HLA-A*02 carriers, elderly individuals had (although not significantly) longer telomeres than the younger ones (elderly group: *n* = 46, telomere length was 8.71 ± 3.79 kb; young group: *n* = 34, 7.16 ± 2.54 kb; *p* = 0.0819; Figure 2Ca). Additionally, HLA-A*02-associated haplotypes were found with increased frequencies among elderly Turks (A*02~C*05, HF young: 0.0095, HF elderly: 0.0410, OR = 4.46, *p* = 0.010; A*02~DRB1*03, HF young: 0.0158, HF elderly: 0.0492, OR = 3.22, *p* = 0.020; Table 3). Furthermore, a slight difference in telomere length was noticed in elderly Turks between HLA-A*02 carriers (*n* = 46) and individuals (*n* = 58) carrying other HLA-A alleles (8.71 ± 3.79 vs. 7.41 ± 3.60 kb, *p* = 0.0873; Figure 2Cc).

Moreover, older Turks carrying HLA-B*18 (*n* = 17; mean telomere length was 9.11 ± 3.72 kb) had longer telomeres than young Turks with this HLA variant (*n* = 13; 6.35 ± 2.26 kb; *p* = 0.0254; Figure 2Cb). In addition, in a group of young Turks, longer telomeres were observed (*n* = 6) in HLA-DRB1*16-positive individuals than in those with other HLA-DRB1 alleles (*n* = 74) (9.36 ± 3.12 vs. 6.74 ± 2.56 kb, respectively, *p* = 0.0154). Of note, Bulgarians carrying HLA-DRB1*16 and/or DRB1*11 also had significantly longer telomeres (Figure 2Ad). A trend in which in the group of elderly people with HLA-DRB1*13 (*n* = 18) had longer telomeres as compared to younger DRB1*13 carriers was observed (*n* = 8; 8.63 ± 4.20 kb vs. 5.89 ± 1.47 kb, respectively; *p* = 0.087).

**Table 3:** Classical HLA haplotypes observed with significantly increased frequency (*p* < 0.05) in elderly and young individuals from the Turkish population.

Haplotype	HF Young (*n* = 158)	HF Elderly (*n* = 122)	OR	CI.L	CI.U	*p*-Value
A*02~C*05	0.0095	0.0410	4.46	1.13	25.42	0.010
B*15~C*03	0.0253	0	0.07	0.00	1.29	0.010
A*01~DRB1*11	0.0253	0.0041	0.16	0.02	1.28	0.048
A*02~DRB1*03	0.0158	0.0492	3.22	1.04	11.8	0.020
A*26~DRB1*04	0.0190	0	0.10	0.01	1.74	0.040
A*26~DRB1*07	0	0.0164	11.84	0.63	221.06	0.040
A*03~DRB1*03	0	0.0164	11.84	0.63	221.06	0.040
B*07~DRB1*15	0.0285	0.0041	0.14	0.02	1.12	0.030
B*51~DRB1*15	0.0285	0.0041	0.14	0.02	1.12	0.030
B*44~DRB1*15	0.0190	0	0.10	0.005	1.74	0.040
C*04~DRB1*04	0.0411	0.0082	0.19	0.02	0.87	0.020
C*04~DRB1*11	0.0222	0.0574	2.69	0.99	7.98	0.030
DRB1*10~DQB1*05	0.0380	0.0082	0.21	0.05	0.94	0.025
DRB1*15~DQB1*05	0.0285	0.0000	0.07	0.004	1.14	0.008
A*03~B*35~DRB1*03	0	0.0164	11.84	0.63	221.06	0.040

HF: haplotype frequency; n: the number of individuals included in this study; OR: odds ratio; CI: confidence interval (95%). *p*-values ≤ 0.05 were considered statistically significant.

In the Polish population, an association between telomere length and the presence of HLA class I and II was detected (Figure 2D). In the context of HLA class I, an association was observed between telomere length and the frequency of HLA-B*15 (Figure 2Da). In young Poles, individuals carrying this allele were characterized by longer telomeres (*n* = 13; 11.87 ± 3.58 kb; *p* = 0.0254; Figure 2Da). Moreover, young Poles carrying HLA-DRB1*01 and DRB1*07 had longer telomeres as compared to the older groups (DRB1*01: mean telomere length was 12.66 ± 4.38 vs. 9.31 ± 3.68 kb, *p* = 0.0221, Figure 2Db; DRB1*07: 11.99 ± 5.15 vs. 9.40 ± 3.85 kb, *p* = 0.0448, Figure 2Dc). Additionally, a tendency was observed in telomere length of healthy elderly Poles carrying or lacking DRB1*13 (Figure 2Dd), where the older group of Poles with HLA-DRB1*13 had longer telomeres compared to subjects carrying other HLA-DRB1 (11.78 ± 3.73 vs. 9.96 ± 3.94 kb, *p* = 0.0544).

Lack of significance in the prevalence of HLA haplotype distribution was observed in elderly Poles when compared to young group. However, some trends toward increased frequencies in elderly were detected for the following haplotypes: B*07~DRB1*07 (HF young: 0, HF elderly: 0.0253, OR: 8.90, *p* = 0.07), B*38~DRB1*15 (HF young: 0, HF elderly: 0.0253, OR: 8.90, *p* = 0.07), B*57~DRB1*07 (HF young: 0.0064, HF elderly: 0.0404, OR: 4.63, *p* = 0.08). Two of them include HLA-B*07 and were found to be related with telomere length.

On the other hand, A*02~B*44 (HF young (*n* = 78): 0.0962, HF elderly (*n* = 99): 0.0404, OR: 0.4, CI.L: 0.14, CI.U: 1.03, *p* = 0.035) and A*11~DRB1*01 (HF young (*n* = 78): 0.0321, HF elderly (*n* = 99): 0, OR: 0.07, CI.L: 0.00, CI.U: 1.26, *p* = 0.016) were found to prevail in young representatives of the Polish population as compared to elderly ones, demonstrating increased frequencies.

## 3. Discussion

Aging is a process that changes the performance of most physiological systems and increases susceptibility to diseases and death [18]. Notably, the World Health Organization has recognized the concept of “healthy aging” as a global priority for modern society [19]. Population aging is a global phenomenon. Virtually every country in the world is seeing an increase in both the number and percentage of elderly people in the population. This is due to declining fertility rates and improved survival rates associated with economic and social development and advances in public health and medicine. Measures and indicators commonly used by the United Nations and other researchers to compare the size of different age groups are based on people’s chronological age and typically define older adults as people who are 60 or 65 years of age or older. Globally, life expectancy at birth is 72.3 years, with women living on average five years longer than men—74.7 years and 69.9 years, respectively [20,21]. Among the populations we studied, Bulgarians had the lowest life expectancy of 73.6 years, while in Poland, the average life expectancy is slightly higher and counts to 78 years. The situation is similar in the Turkish population. The average life expectancy of Turks is approximately 79 years. Importantly, among the populations we study, Turkey (+24 years) has seen the largest increase in life expectancy since 1970. For Romanians, the average life expectancy is approximately 75 years.

Telomeres are a major compartment of aging, and their length has been correlated with measures of health and fitness in elderly adults [22]. Results from the Louisiana Healthy Aging Study confirmed that telomere length correlates with measures of healthy aging in an age-dependent manner [23]. Ashkenazi centenarians and their offspring showed longer telomeres, for their age, compared to controls, and longer telomeres correlated with fewer diseases [24]. In contrast, in a study of Canadian “Super-Seniors” (people 85 years of age or older who had not been diagnosed with any disease or cancer), the healthy oldest-old did not have exceptional telomere length for their age but showed less variability in telomere length than middle-age controls, suggesting that they may be selected for optimal rather than extreme telomere length [25]. This is confirmed by the research of Arai et al., where the average telomere length decreased in centenarians (to app. 3.5 kb) and then remained more or less unchanged or, paradoxically, even increased in supercentenarians compared to younger age groups [26]. This evidence suggests that centenarians can better maintain telomere length and telomerase activity than non-centenarians and that healthy centenarians have longer telomeres than unhealthy ones [27,28,29].

In the present study, we demonstrated differences in telomer length between the four populations studied, as well as between their young and the elderly representatives. Our results show some interpopulation differences in telomere length. Among the elderly individuals, Poles had the longest telomeres. A significant difference in telomere length was observed between healthy elderly Bulgarians and Turks. In young study groups, Romanians had the longest telomere length. The presented study is the first to report on HLA diversity and HLA relationships with telomere length in representatives of four different populations—Bulgarians, Romanians, Turks, and Poles. So far, many studies have investigated the possible association between HLA and human longevity, but no research has been conducted on the relationship between HLA, telomere length, and longevity.

The role of genetics in determining longevity is complex. It appears that MHC may play an important role in age-related immunological changes, since most diseases observed with aging have an immunological pathogenesis. Complex immunological features associated with lifelong immune reactivity and immunosenescence, such as circulating immunoglobulin levels, peripheral CD4/CD8 T lymphocyte ratio, and telomere length, appear to be under tight genetic control [18,30,31].

HLA gene frequencies show a high degree of variability between populations and a striking geographical correlation. These frequencies are useful for inferring the genetic origins and ethnic structure of modern human groups [32]. For example, this relationship was seen in the elderly Greek population, where HLA-B16 and HLA-DR7 were the most common, while HLA-B15 and HLA-DR4 were found with lower frequency in the study group [33]. In elderly Romanians, HLA-A*01:01:01:01 and HLA-DRB1*07:01:01:01 were some of the most common HLA alleles [34]. Moreover, alleles and haplotypes associated with autoimmunity were not observed in families with long-lived members. This is consistent with the hypothesis that life extension is related to a delay in immunodeficiency of normal aging or the possible improvement in autoimmunity that develops with age [35]. The implementation of high-resolution DNA typing methods has enabled the detection of a much more comprehensive spectrum of variation, including rare alleles and haplotypes, and has made HLA comparisons a discriminating tool for further elucidation of population relationships [36].

Evidence for the link between telomere length and HLA genes is scarce, but so far HLA-DRB1*04 alleles have been associated with excessive telomere in CD4+ T cells. Therefore, it has been proposed that HLA-DRB1*04 alleles or genes, in the event of linkage disequilibrium, regulate stem cell replication and contribute to the generation of senescent and autoreactive T cells [37]. Interestingly, in Japanese people from Okinawa, known for their longevity, it was observed that the frequencies of HLA-DRB1*14:01, DQB1*05:03, DQA1*01:01, and 01:04, DQA1*05 were increased in centenarians. Further, the frequencies of DRB1*01:01 and DRB1*12:01 have tended to be associated with longevity [38]. Our study showed that HLA-DQA1*01 is associated with longer telomeres in the elderly Romanian population, and furthermore, HLA-DQA1*01-associated haplotype was observed at an increased frequency in elderly Romanians. Additionally, we observed strong relationships between HLA-DRB1*11 and/or DRB1*16 with telomere length in the Bulgarian population. Interestingly, DRB1*11- and DRB*16-associated haplotypes were previously found by Naumova et al. with statistically significant increased frequencies in elderly individuals from the Bulgarian population when compared to the young group [35]. Similarly, in the present study, DRB1*11- (A*01~DRB1*11) and DRB1*16 (A*02~B*35~DRB1*16)-associated haplotypes were found to significantly prevail among elderly Bulgarians. Among Poles, DRB1*13, *16, *01, and *07 were found to be associated with longer telomeres. We also observed some trends in elderly Poles regarding increased frequencies of two B*07-associated haplotypes (B*07~DRB1*07 and B*57~DRB1*07). A study conducted by Ivanova et al. showed a relationship between HLA-DRB1*07 and longevity in the Greek and French populations [39]. So far, it has been described that HLA-DRB1*11 alleles and haplotypes are associated with protection against autoimmune diseases and HCV and HBV infections in southern European populations [40]. Also, it is well known that HLA-DRB1*13 has a protective effect on infectious diseases. A more recent study has shown that the DRB1*13:02, DRB1*14:01, and DRB1*16:02 haplotypes have a clear correlation with life span [41]. Previous research in accordance with this study suggest that human longevity may be related to telomere length, associated with several alleles of the HLA-DRB1 and/or HLA-DQ loci.

To summarize, in this study, we demonstrated the differences in telomere length between Bulgarians, Romanians, Turks, and Poles, as well as significant associations of the effect of aging on telomere length in two of four analyzed populations. As expected, elderly Bulgarians and Romanians were characterized by significantly shorter telomeres than young representatives of their respective populations. Surprisingly, however, no such relationship was found in Turks and Poles. These unexpected observations can be attributed to differences in mean age within each group analyzed, which was particularly evident in the case of individuals from Turkey (Table 3). Elderly Turks were approximately ten to fourteen years older than elderly representatives from other countries. Similarly, younger Turks were also older than other younger subjects investigated. Moreover, the difference in average age between older and younger individuals in a given population was higher for Turks and amounted to 47 years. We also demonstrated the differences in haplotype segregation between the young and the elderly representatives of all studied populations. Interestingly we also found some significant relationships between telomere length and HLA type/haplotype in the study groups. After many years of longevity research, it is known that this process is associated with the positive or negative selection of alleles and haplotypes that confer resistance or susceptibility to disease, respectively, through peptide presentation or through nonspecific antigen control of the immune response [30].

To the best of our knowledge, the present work is the first population-based study to elucidate the associations of HLA with telomere length and longevity. Understanding the factors that determine longevity can help extend healthy lives for a wider population and close the gap between the fastest- and the slowest-aging population groups. Knowledge about the biology of telomere length continues to expand, and their impact on the aging process and occurrence of age-related diseases seems to be important. Because the trajectories of aging begin to diverge in early adulthood [42], it is critical to assess the contribution of telomere-related changes to the diverse aging pathways. The observed correlations between HLA and telomere lengths support the association of HLA genes with the longevity process. Nevertheless, further, more extended studies are needed to explain the biological basis of the observed relationships.

## 4. Materials and Methods

### 4.1. Subjects

Within the 18th IHIWS, a total of 957 samples from four populations (Polish, Bulgarian, Romanian, and Turkish) were collected, where two main data sets were analyzed: unrelated, healthy elderly individuals (*n* = 392, age 65–99 years) and an ethnically matched young group (*n* = 565; age 18–64 years). Healthy elderly individuals were characterized according to the SENIEUR protocol, and young representatives according to the JUNIEUR protocol [43]. All samples were previously genotyped for HLA alleles using various sequencing methodology. The analyses were performed in the respective national, EFI accredited HLA typing laboratories (EFI—European Federation for Immunogenetics). For the purpose of this study, low-resolution data and haplotype analysis were employed. The characteristics of the study groups are presented in Table 4.

### 4.2. Quantification of Telomere Length

Mean telomere length was measured in genomic DNA samples of 927 unrelated healthy individuals, provided by the participating laboratories/centers. To reduce and exclude the possibility of laboratory errors, the entire telomere length analysis of all samples was carried out by the same person in one laboratory in Poland. The DNA samples were diluted with nuclease-free water to a concentration of 5 ng/mL. DNA concentration and purity were quantified by DeNovix DS-11 spectrophotometer (DeNovix Inc., Wilmington, DE, USA). Telomere length measurements were performed on a LightCycler480 II Real-Time PCR system (Roche Diagnostics International, Rotkreuz, Switzerland) using qPCR test kits (ScienCell’s Absolute Human Telomere Length Quantification qPCR Assay Kit [AHTLQ], Carlsbad, CA, USA), as previously described by Dratwa et al. [44]. The PCR conditions were as follows: 95 °C for 10 min followed by 32 cycles of 95 °C for 20 s, 52 °C for 20 s, and 72 °C for 45 s. Data analysis was conducted according to the manufacturer’s instructions. All reactions were run in three replicates.

### 4.3. Statistical Analysis

In each experiment, the normality of data was verified with the Shapiro–Wilk test. Statistical analyses for the assessment of differences between groups were performed using one-way analysis of variance (ANOVA) and the obtained *p*-values were corrected by the Benjamini and Hochberg method. In cases where the distribution of data deviated from the normal distribution, the non-parametric Mann–Whitney U test was performed for the comparison of telomere lengths. The statistical calculations were performed using GraphPad Prism software (GraphPad Software, La Jolla, CA, USA, version 8.0.1) and the Real Statistics Resource Pack for Microsoft Excel 2019 (version 16.0.10369.20032, Microsoft Corporation, Redmont, Washington, DC, USA). The probability (*p*) values < 0.05 were considered statistically significant, while the trend index was between 0.05 and 0.10. For haplotype analysis and confidence interval, odds ratio, and *p*-value estimation, the Bridging ImmunoGenomic Data-Analysis Workflow Gaps (BIGDAWG) data analysis pipeline was used [45].

## Figures and Tables

**Table 4:** Characteristics of the study groups.

	Elderly Individuals	Young Individuals
Bulgaria	N = 68	N = 121
Male, n (%)	24 (35%)	43 (36%)
Female, n (%)	44 (65%)	78 (64%)
Mean age (years ± SD)	71 ± 6.19	27 ± 4.03
Romania	N = 100	N = 100
Male, n (%)	49 (49%)	55 (55%)
Female, n (%)	51 (51%)	45 (45%)
Mean age (years ± SD)	71 ± 5.74	30 ± 3.40
Turkey	N = 125	N = 80
Male, n (%)	38 (30%)	38 (48%)
Female, n (%)	87 (70%)	42 (52%)
Mean age (years ± SD)	85 ± 6.99	37.79 ± 6.44
Poland	N = 99	N = 264
Male, n (%)	37 (37%)	134 (51%)
Female, n (%)	62 (63%)	130 (49%)
Mean age (years ± SD)	74 ± 6.19	34 ± 9.06

## Data Availability

The data presented in this study are available upon request from the corresponding author. The data are not publicly available due to privacy or ethical restrictions.
